# Draft Genome Sequence of a Multicountry Outbreak-Related Listeria monocytogenes Sequence Type 1247 Strain, VLTRLM2013

**DOI:** 10.1128/MRA.00698-20

**Published:** 2020-08-06

**Authors:** Mihkel Mäesaar, Mati Roasto

**Affiliations:** a Chair of Food Hygiene and Veterinary Public Health, Institute of Veterinary Medicine and Animal Sciences, Estonian University of Life Sciences, Tartu, Estonia; University of Maryland School of Medicine

## Abstract

We sequenced the genome of a multicountry outbreak-related Listeria monocytogenes sequence type 1247 strain (VLTRLM2013) that was isolated from a vacuum-packaged sliced salted salmon product of an Estonian fish-processing company that was obtained from an Estonian retail outlet in 2013.

## ANNOUNCEMENT

Ready-to-eat (RTE) fish products from retail outlets have higher prevalence and counts of Listeria monocytogenes than any other RTE food category in Estonia (1). RTE fish products that have not been listericidally treated are often the cause of listeriosis outbreaks (2). An analysis of strains isolated from a prolonged multicountry outbreak identified sequence type 1247 (ST1247) as the main cause of 22 listeriosis cases in the European Union in 2014 to 2019 (3).

L. monocytogenes strain VLTRLM2013 was isolated in accordance with Estonian Centre for Standardisation standard EVS-EN ISO 11290-1:2000/A1:2004 (4) and stored in an ultra-low freezer (Sanyo Electric Company, Japan) at −82°C in Protect microorganism preservation system tubes (Technical Service Consultants, UK) in the laboratory of the Chair of Food Hygiene and Veterinary Public Health of the Estonian University of Life Sciences. In 2020, the strain was recultivated on blood agar (Biolife Italiana, Italy) at 37°C under aerobic growth conditions. Genomic DNA extraction was preceded by pretreatment with a Precellys 24 homogenizer using a lysing kit CK01 tube (Bertin Instruments, France) and buffer ATL (Qiagen, Netherlands). DNA was extracted using the IndiSpin pathogen kit (Indical Biosciences, Germany), and the concentration was measured using a Qubit 4 fluorometer with the double-stranded DNA (dsDNA) broad-range assay kit (Thermo Fisher Scientific, USA). DNA was sheared using an M220 focused ultrasonicator (Covaris, USA) and used to prepare the library using the TruSeq DNA PCR-free library preparation kit (Illumina, USA). The aforementioned procedures were performed according to the manufacturers’ recommendations. DNA extraction and sequencing were performed at the Estonian Veterinary and Food Laboratory using a MiSeq system with the MiSeq reagent kit v3 (600 cycles; Illumina), producing 425,773 pairs of reads with a total length of 250.67 Mbp.

Raw and trimmed read quality was checked using FastQC v0.11.9 (5), and reads were trimmed using Trimmomatic v0.39 (6) (ILLUMINACLIP:<adapter_sequences>:2:30:10:1:true LEADING:3 TRAILING:3 SLIDINGWINDOW:4:20 HEADCROP:5 CROP:295 MINLEN:36). Trimming resulted in 410,917 pairs of reads, with a length of 227.50 Mbp, which were assembled into an initial draft genome with SPAdes v3.14.1 (7) using the parameter --careful. Four filtering steps were then performed as described by Llarena et al. (8). Contigs with a length of <200 bp, k-mer coverage of <2×, GC content of <5% or >95%, and coverage less than one third of mean draft genome coverage were excluded. For the reference-mapping approach using BWA v0.7.17-r1188 (9) index, mem, and SAMtools v1.10 (10) view, sort commands were applied to map trimmed reads to the filtered draft genome assembly. Results were assessed using the Qualimap v2.2.1 (11) bamqc command. BLAST (12) was used to detect possible contaminant sequences in all of the contigs included in the filtered draft genome assembly. Multilocus sequence typing (MLST) was performed using mlst v2.18.0 (13, 14).

The reported L. monocytogenes ST1247 strain VLTRLM2013 draft genome is 2,924,666 bp, assembled in 17 contigs with an *N*_50_ value of 579,564 bp, a GC content of 38%, and coverage of 77.74×. The draft genome was annotated using the NCBI Prokaryotic Genome Annotation Pipeline (PGAP) v4.11 (15) with default settings. The annotated draft genome contained 2,855 protein-coding genes.

### Data availability.

This whole-genome shotgun project has been deposited in GenBank under the accession no. JABGCT000000000. The version described in this paper is the first version, JABGCT010000000. The raw sequencing reads have been deposited in the SRA under the accession no. SRR11789329.
